# EEG time-frequency dynamics of early cognitive control development

**DOI:** 10.1016/j.dcn.2025.101548

**Published:** 2025-03-22

**Authors:** Santiago Morales, George A. Buzzell

**Affiliations:** a Department of Psychology, University of Southern California, Los Angeles, CA, USA; b Department of Psychology, Florida International University, Miami, FL, USA; c Center for Children and Families, Florida International University, Miami, FL, USA

**Keywords:** EEG, Cognitive control, Time-frequency, Theta, Neural oscillations, development

## Abstract

Cognitive control is crucial for goal-directed behavior, and essential for other aspects of cognitive and socioemotional development. This review examines when and how the neural dynamics of cognitive control emerge and develop, focusing on electroencephalography measures used to study cognitive control in infants and children. We argue that time-frequency analyses are uniquely able to capture two distinct components of cognitive control: 1) the detection that control is needed, and 2) the instantiation of control. Starting in infancy and increasing across childhood and adolescence, studies suggest the signal strength and consistency of midfrontal theta and delta oscillations are involved in processes that detect the need for control. For control instantiation, there is evidence that theta band connectivity between midfrontal and lateral-frontal cortices is present from early childhood. There is also evidence for the involvement of midfrontal theta power in the instantiation of control in infancy. We further review emerging evidence that indicates individual differences in midfrontal theta are not only proximally related to behavior, but also sensitive to variations in early experience and risk for psychopathology, providing a neural mechanism linking early adversity to future psychopathology. We discuss needed future steps, including novel paradigms, computational models, and aperiodic/periodic modeling of EEG.

## 1 Introduction

Cognitive control^2^ is often defined as the ability or set of cognitive processes that allow for making adaptive changes to behavior and cognition to achieve task goals (Gratton et al., 2018, Nigg, 2017). Moreover, cognitive control can be defined as involving at least two general components: 1) the detection that control is needed (e.g., detecting errors, conflict, changing task demands), and 2) the instantiation of control to achieve goal-directed behavior (e.g., increasing attentional focus or inhibiting unwanted responses). Cognitive control is known to emerge and undergo significant development from infancy to early childhood, as cognitive control first emerges during early infancy and transitions from mostly being driven by exogenous forms of control (e.g., reliant on the caregiver) to more endogenous and complex forms of control (e.g., goal-driven performance monitoring and planning) (Kopp, 1982, Morales and Fox, 2019, Munakata et al., 2012). Cognitive control is considered to continue developing through adolescence and into early adulthood, but these changes are thought to be more gradual (Luna et al., 2004, Ordaz et al., 2013, Zelazo et al., 2013).

Given that cognitive control plays a central role in goal-directed behavior, cognitive control is also essential for other aspects of cognitive and socioemotional development (Morales and Fox, 2019, Rueda et al., 2010). This is evidenced not only by concurrent associations, but also longitudinal relations with future psychopathology, academic achievement, social competence, financial success, criminal offending, and health outcomes (Mischel et al., 1989, Moffitt et al., 2011, Morales et al., 2016, Morales et al., 2020, Robson et al., 2020). Given the developmental importance of cognitive control, a better understanding of when and how the neural dynamics of cognitive control emerge and develop is of paramount importance. Examining the neural mechanisms of cognitive control in early childhood has several notable implications. First, and at a fundamental level, it can provide important insights into how neural development gives rise to the emergence of increasingly endogenous and sophisticated forms of cognitive control, beginning in infancy and toddlerhood. Second, by increasing our understanding of cognitive control and establishing new ways of capturing the neural mechanisms underlying cognitive control in early development, it might be possible to identify early neural markers of risk for later developmental difficulties, even before such differences are evident in behavior. Third, such markers could also serve as possible targets for brain-based intervention approaches.

In the current review, we first provide a basic introduction to different electroencephalography (EEG) approaches that can be employed to study cognitive control in infants and children. We place a special emphasis on time-frequency measures and argue that they are uniquely positioned to provide crucial information regarding neural mechanisms involved in cognitive control across infancy and childhood. We then review emerging studies from our group and others that utilize EEG-based measures to study cognitive control, emphasizing the utility of time-frequency analyses to detect developmental phenomena unobservable via other approaches. In reviewing these studies, we place special emphasis on findings that involve theta oscillations, given that theta is thought to play a central role in cognitive control (Cavanagh and Frank, 2014). As an organizing framework and at a heuristic level, we categorize the review of studies within the context of the Detection and Dual Control (DDC) framework (Fox et al., 2021, Fox et al., 2022). Finally, we discuss future directions to better understand the neural mechanisms of cognitive control and its development, especially during the first years of life.

## 2 EEG time-frequency approaches provide unique information regarding neurocognitive development

EEG is one of the most efficient and accessible methods for studying brain function across development and is commonly employed to study cognitive control. EEG offers several distinct advantages over other neuroimaging methods (Buzzell et al., 2023). These advantages include a high temporal resolution that matches the speed at which psychological processes occur, allowing measurement of cognitive dynamics in real time (Cohen, 2014b, Luck, 2014). Additionally, EEG directly measures brain activity, with the voltage fluctuations recorded at the scalp reflecting the direct readout of biophysical phenomena at the neuronal population level (Buzsáki et al., 2012, Cohen, 2014b, Luck, 2014). EEG places a relatively low burden on participants and is a highly adaptable method of measuring functional brain activity across the lifespan, including from awake and behaving infants. EEG is also relatively inexpensive and portable compared to other brain imaging techniques, facilitating mobile and bedside data collection (e.g., Troller-Renfree et al., 2021). Crucially, mobile EEG can be utilized in a wide variety of settings and with populations for whom attending the laboratory may be difficult, ultimately leading to more inclusive data collection and enhanced demographic representation in EEG research. Finally, the fact that EEG is already present in many clinics/healthcare centers means that clinically-relevant research discoveries can be rapidly implemented at scale, given the necessary infrastructure is already in place (Cavanagh, 2019).

Despite the advantages of EEG, the majority of published developmental research continues to use a narrow number of EEG data collection and analysis approaches, limiting the knowledge gained (Buzzell et al., 2023). For example, in a recent literature review of all EEG manuscripts published in *Developmental Cognitive Neuroscience,* we found that most papers (approximately 77 %) used only event-related potential (ERP) analyses (Morales and Bowers, 2022). This was followed by what we called Fourier-based power methods—studies in which oscillation power was analyzed without considering time dynamics. However, only ∼4 % of manuscripts utilized time-frequency analyses of EEG (Morales and Bowers, 2022). Thus, even though EEG time-frequency analyses have been well-established for decades and are more widely used in the cognitive neuroscience literature with adults (Delorme and Makeig, 2004), time-frequency analyses are rarely used to study pediatric EEG data. This is true regardless of previous calls outlining the benefits of time-frequency analyses in studying developmental processes (Bishop et al., 2011, Maguire and Abel, 2013).

Although the ERP technique can provide important insights into neurocognition, this approach yields only a fraction of the knowledge that can be gleaned from EEG data. One limitation of using ERPs to examine developmental processes is that the ERP analysis technique is only sensitive to neural effects that are time-locked to events of interest and temporally consistent across trials. Within the context of ERP analyses, neural effects that are not time-locked are assumed to reflect noise and are “averaged out” when computing trial-averaged ERPs (Luck, 2014). To illustrate this, in Morales and Bowers (2022), we simulated two neural effects (time-domain voltage changes) at the trial level, one occurring at 0 ms and the other at 500 ms. The first neural effect at 0 ms was simulated to vary slightly in its latency across trials, reflecting reduced temporal consistency across trials. As a result of the first neural effect not being precisely time-locked to the event of interest, this effect is averaged out as noise and does not appear in the trial-averaged ERP plot. In contrast, the second neural effect at 500 ms was simulated to have perfect temporal consistency across trials (i.e., perfectly time-locked). As a result, this second neural effect is retained when averaging across trials and is clearly present in the trial-averaged ERP plot. Although this simulated example is hypothetical, it nonetheless illustrates why ERPs are unable to capture neural effects that are not temporally synchronous (time-locked) across trials. Crucially, increasing evidence demonstrates examples of neural effects linked to cognitive control that are not precisely time-locked to events of interest (e.g., Cohen and Donner, 2013). Moreover, several studies have found that the temporal consistency of at least some neural effects increases with age (DuPuis et al., 2015, Gavin et al., 2019, Morales et al., 2022). The ERP technique alone is unable to capture such variability; however, time-frequency approaches can fill this gap.

**Fig. 1:**
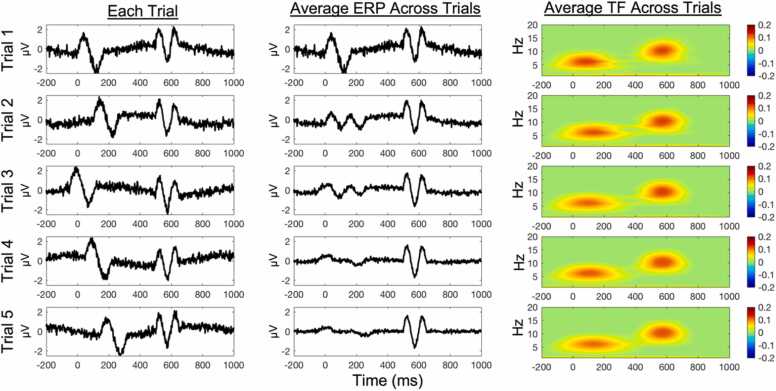
ERP and time-frequency analysis comparison. The left column depicts a simulated EEG signal across five trials. Trials were simulated to have a non-phased-locked 6 Hz response around 0 ms and a phase-locked 10 Hz response around 500 ms. The middle column shows the running average ERP of each trial and all previous trials. The third column shows the running average time-frequency power of each trial and all previous trials. ERP = Event-Related Potential. TF = Time-Frequency. Figure reprinted from Morales and Bowers (2022), licensed under CC BY-NC-ND 4.0 (Creative Commons License)

**Fig. 2:**
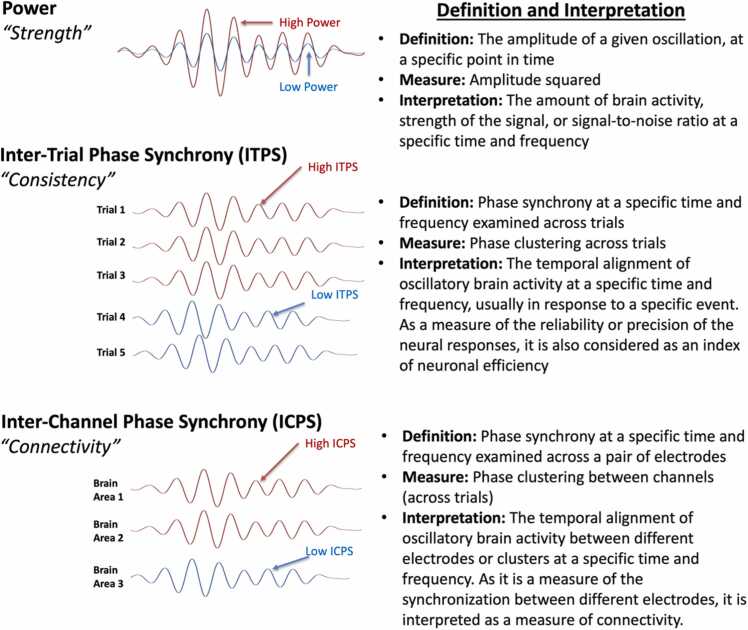
Key time-frequency concepts. Rows correspond to three common time-frequency concepts: power, inter-trial phase synchrony (ITPS), and inter-channel phase synchrony (ICPS), from the top to the bottom row, respectively. The left column provides labels and illustrations of each concept based on simulated data. The right column provides definitions, measures, and general interpretations of each time-frequency concept. Note: It is also possible to compute measures of synchrony (either within or between electrodes) across time instead of across trials. However, the current review focuses only on measures of synchrony computed across trials. When synchrony is computed at a single location, it is referred to as ITPS; when synchrony is computed across locations, it is referred to as ICPS.

To better capture neural effects that are not precisely time-locked to events of interest, one possibility is to conceptualize and measure brain activity as time-varying oscillations (i.e., time-frequency representations) rather than as averaged time-varying changes in raw voltage amplitudes (i.e., ERPs). Beyond the theoretical benefits that we describe later, characterizing EEG as oscillations provides several measurement advantages, allowing the capture of neural effects that are not precisely time-locked to events of interest (see Fig. 1) and providing a metric that separately quantifies the degree of temporal consistency (see Fig. 2). Briefly, oscillations are described by several independent features (frequency, amplitude, and phase) that yield additional possibilities for characterizing neural dynamics. The frequency of an oscillation defines how long in time it takes to complete one full cycle (i.e., how “fast” the oscillation is) and is measured in terms of cycles per second as Hertz (Hz). Amplitude refers to the distance between the zero point of the oscillation and the highest/lowest points (i.e., the “magnitude” of the oscillation). Phase refers to the position along an oscillation’s cycle that aligns with a given point in time (i.e., the “alignment” of the oscillation). For EEG analyses of oscillations, one commonly computed measure is power (Cohen, 2014b), which is defined as the amplitude squared for a given frequency (see Fig. 2). In general terms, power can be conceptualized as the “strength” of a given oscillation. To date, most developmental studies have investigated event-related power dynamics by averaging power within relatively narrow frequency bands, such as within the theta band. However, it is also possible to model and quantify broader, underlying (aperiodic) patterns in the power spectrum across a wider range of frequencies. Specifically, recent approaches can quantify the overall (1/f-like) slope of the power spectrum, as well as the overall offset of the power spectrum, corresponding to mean levels of broadband power (e.g., Donoghue et al., 2020). In the “Recommendations for Future Research” section, we discuss the methodological and theoretical utility of these approaches, as well as the need for studies applying these approaches within development samples.

It is also possible to measure the degree to which the phase of a given oscillation frequency aligns or synchronizes across trials, either within a given electrode or across electrodes (see Fig. 2). Phase alignment/synchrony at a given electrode (across trials) is often referred to as inter-trial phase synchrony (ITPS; Cohen, 2014b) and can be conceptualized heuristically as the “consistency” of given oscillation across trials. In contrast, phase alignment/synchrony between two electrodes (across trials) is often referred to as inter-channel phase synchrony (ICPS; Cohen, 2014b) and is interpreted heuristically as a measure of “connectivity” (i.e., reflecting neural communication) between two brain regions. Although beyond the scope of the current review, it is also possible to compute phase alignment across time, as well as measure inter-relations of phase or power within or across frequencies; the reader is referred to Cohen (2014b) for further details.

Returning to our simulated EEG example of time domain voltage changes at the trial level, we can represent these same signals as time-by-frequency plots, with the x-axis denoting time, the y-axis denoting frequency, and power denoted along the z-axis as color, such that warmer colors indicate more power and cooler colors indicate less power (see Fig. 1). The time-frequency representation reveals that the neural effect happening at 0 ms reflects a 6 Hz “theta” response, and the neural effect at 500 ms reflects a 10 Hz “alpha” response. Importantly, we can see that both the early (0 ms) and late (500 ms) neural effects are represented in the trial-averaged time-frequency plot of power, despite the lack of temporal consistency for the earlier component across trials (see Fig. 1). Thus, time-frequency representations of power are relatively robust to temporal variability across trials. In this way, some time-frequency measures are more forgiving of imprecise paradigm timing than ERPs, making them particularly useful for infant studies given that some employ behavioral coding, which involves an inherent lack of timing precision (e.g., Debnath et al., 2019).^3^ We can further employ ITPS to measure the phase alignment across trials to directly quantify the level of temporal consistency. In so doing, we find that the early component has an ITPS value of.23 within the 6 Hz frequency band (relatively low consistency) and the later component has an ITPS value of 1 (perfect consistency) within the 10 Hz frequency band. Thus, time-frequency analyses are not only able to capture neural effects that are not precisely time-locked, but also yield independent measures that depict the power (strength) and synchrony (consistency) of a given neural effect.

In addition to the measurement advantages described above, EEG time-frequency analyses have several additional benefits at both the theoretical and practical levels. A major strength of time-frequency analyses over other EEG methods is their interpretability. Because neuronal oscillations are a fundamental property of the brain (Buzsaki, 2004), time-frequency measures provide more direct information regarding the neurophysiological mechanisms underlying the processes captured by EEG data (Cohen, 2014b). Moreover, oscillatory activity is considered to be causally implicated in cognition (Cavanagh and Frank, 2014, Herrmann et al., 2016, Klimesch, 2012, Narayanan et al., 2013), as discussed in the subsequent section. EEG time-frequency measures also provide a bridge to multiple disciplines of neurophysiology (e.g., single-cell recordings, nonhuman animal work, intracranial EEG, and MEG; (Buzsáki et al., 2012, Cavanagh et al., 2021).

Having provided a basic primer on EEG time-frequency analysis, this review seeks to highlight the utility of time-frequency analyses for understanding the development of cognitive control. In particular, we review examples of studies that employed EEG time-frequency analyses to elucidate developmental processes that may have otherwise been missed via the more commonly employed ERP technique. Our review emphasizes the role of theta oscillations, given mounting evidence suggesting theta oscillations play a mechanistic role in cognitive control (Cavanagh and Frank, 2014). In the subsequent section, we briefly introduce the proposed role of theta in cognitive control.

## 3 Time-frequency EEG can index a possible cognitive control mechanism: midfrontal theta

Mounting evidence suggests brain oscillations play a mechanistic role in various aspects of behavior and cognition (Buzsáki et al., 2012, Cavanagh and Frank, 2014, Fries, 2005, Herrmann et al., 2016, Lisman and Jensen, 2013). In particular, event-related^4^ theta band oscillations recorded over midfrontal regions have been proposed as a candidate mechanism through which the brain both detects the need for control, as well as implements control by coordinating activity across brain networks (Cavanagh and Frank, 2014). The proposed role of midfrontal theta in cognitive control is consistent with other work demonstrating that slower oscillations like theta are well-suited to facilitate the maintenance, manipulation, or communication of information across brain regions (Buzsaki, 2004, Buzsáki et al., 2012, Helfrich and Knight, 2016, Lisman and Jensen, 2013). Moreover, imaging work finds that medial-frontal cortex activity is closely associated with the detection that control is needed, whereas coordinated brain activity between the medial-frontal cortex (MFC) and frontal-lateral cortices is associated with the instantiation of control (Kerns et al., 2004, MacDonald et al., 2000, Ridderinkhof et al., 2004). Consistently, midfrontal theta oscillations—likely arising in part from midcingulate cortex—are enhanced in situations involving the detection that control is needed (Cavanagh and Frank, 2014). Similarly, synchrony of theta oscillations at electrode locations located over MFC and frontolateral regions is associated with behavioral changes indicative of control instantiation (Cavanagh and Frank, 2014). Evidence consistent with the proposed role of midfrontal theta in cognitive control comes from both noninvasive (e.g., Buzzell et al., 2019; Cavanagh et al., 2009) and invasive (e.g., Voytek et al., 2015) recordings in humans, as well as electrophysiological recordings in animal models (e.g., Narayanan et al., 2013; Tsujimoto et al., 2006). Moreover, causal evidence for the role of midfrontal theta in cognitive control has been demonstrated via stimulation work (Grover et al., 2021, Miller et al., 2015, Reinhart, 2017, Reinhart et al., 2015).

Integral to the notion that midfrontal theta plays a mechanistic role in cognitive control is prior work demonstrating that slower oscillations (like theta) can provide a temporal structure that simultaneously supports the integration and segregation (multiplexing) of information within and between overlapping, distributed brain networks (Cavanagh and Frank, 2014, Cohen, 2014a, Duprez et al., 2020, Helfrich and Knight, 2016). Brain oscillations reflect temporal patterns of alternating excitation/inhibition of the local field potential (LFP), which produce temporal windows of increased excitation/inhibition in which neurons are more/less likely to fire (Buzsaki, 2004, Buzsáki et al., 2012). Thus, precise temporal synchronization across a network of brain regions aligns windows of excitation/inhibition, facilitating selective exchange of information and shielding against interference (Fries, 2005). The phase of oscillations further allows for the presence of overlapping networks in which the same cortical region participates in multiple distal brain networks—effectively serving as a “hub”—by synchronizing at distinct phases (Duprez et al., 2020, Lisman and Jensen, 2013). Relatedly, slower oscillations (e.g., theta) are particularly well-suited for synchronization across distal cortical regions, given that the temporal structure of slower oscillations is better maintained over longer distances (Buzsaki, 2004, Buzsáki et al., 2012).

The temporal structure provided by midfrontal theta may describe the mechanistic role of midfrontal theta in cognitive control (Cavanagh and Frank, 2014, Cohen, 2014a, Duprez et al., 2020, Helfrich and Knight, 2016). As such, developmental changes in midfrontal theta may provide the enhanced temporal precision necessary to maintain multiple, overlapping networks involving the MFC, with reduced interference and improved information exchange, yielding the emergence of improved and more complex forms of cognitive control behavior. Of note, known developmental changes in brain structure (e.g., gray and white matter) are known to coincide with developmental changes in the synchrony of oscillations (Uhlhaas et al., 2010). Ultimately, studying the development of midfrontal theta provides the opportunity to move beyond “markers” of cognitive control, towards understanding developmental changes in a fundamental mechanism that allows for the emergence of improved cognitive control behavior. Formalized computational models describing the precise role of midfrontal theta in cognitive control within a developmental context are needed (see Recommendations for Future Research). Nonetheless, this review provides a survey of existing findings on age-related changes in midfrontal theta that can provide the foundation to develop and ultimately test such models.

In studying oscillations across development, it is important to note that the frequency bands of oscillatory processes exhibit a shift across infancy and into childhood (Saby and Marshall, 2012), such that “theta” is often defined as 3–5 Hz in infants and 4–8 Hz in children and adults. Although approaches for individualizing the selection of frequency ranges exist (e.g., see Donoghue et al., 2020; Klimesch, 1997), these are less commonly employed in the literature. Similarly, although emerging work (Donoghue et al., 2020) suggests power within narrow frequency bands may at least partially be explained by broader patterns across a wider range of frequencies (i.e., aperiodic slope and offset), these approaches have not yet been deployed to study the development of event-related power in cognitive control tasks. As such, in the current review, we focus on analyses of narrowband power, relying on the theta band definitions provided by each reviewed study. Where appropriate, we also report related findings associated with the “delta band” (< 3–4 Hz). However, before turning to a review of the developmental cognitive control literature, the next section briefly introduces the Detection and Dual Control (DDC) framework, which we use to organize our review of studies reporting ERP and time-frequency assessments of cognitive control across development.

## 4 The Detection and Dual Control Framework as a useful heuristic for describing cognitive control development

**Fig. 3:**
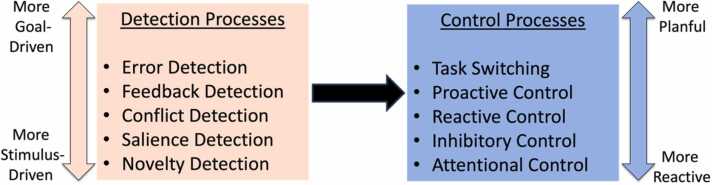
A simplified version of the Detection and Dual Control framework. The boxes and colors indicate the categorization of cognitive control processes into the two broad categories of detection and control. Detection processes (left box) range continuously from more stimulus-driven (bottom) to more goal-driven (top). Control processes (right box) range continuously from more reactive (bottom) to more planful (top). Figure adapted from Fox et al. (2021).

Along with our colleagues (Fox et al., 2021, Fox et al., 2022), we recently proposed a heuristic framework useful for categorizing cognitive control processes called the Detection and Dual Control framework. The DDC framework was originally created to describe and categorize how different components of cognitive control influence relations between early individual differences in infant temperament and later behavioral and socioemotional outcomes (Fox et al., 2021, Fox et al., 2022). Consequently, in the current review, we do not intend to utilize the DDC framework as a comprehensive framework for understanding cognitive control, nor should the framework be interpreted as a model of cognitive control. Nonetheless, we find that a simplified version of the DDC framework (see Fig. 3) is useful for categorizing and describing various processes involved in cognitive control, especially within a developmental context.

Consistent with other frameworks that describe cognitive control (for a review, see Gratton et al., 2018; Nigg, 2017), the DDC framework describes cognitive control as involving two broad components: 1) detection processes that are involved in the realization that control is needed (e.g., novelty/salience detection, errors/conflict monitoring), and 2) control processes to instantiate needed changes to cognition and behavior (e.g., attention/inhibitory control, task switching). The distinction between detection and control processes is indicated in Fig. 3, with detection processes shown on the left and control processes on the right. Detection processes can be conceptualized as an “alarm” that captures an individual’s attention, interrupts automatic behaviors, and provides an alert that controlled responding is required. Crucially, the DDC framework notes that some detection processes are more driven by external stimuli (e.g., novelty detection), whereas others are more strongly driven by internal goals (e.g., error detection). As indicated in Fig. 3, the differences between stimulus-driven and goal-driven detection lie on a continuum rather than being qualitatively distinct. Once the need for control is realized, control processes are involved in the actual instantiation of controlled responding through modifications to attention, inhibition, or updating working memory and/or task rules. The DDC framework also builds on the Dual Mechanisms of Control framework (Braver, 2012) and distinguishes between more reactive and more planful control instantiation processes (Fox et al., 2021, Fox et al., 2022), again indicated as a continuum in Fig. 3. In the remainder of the manuscript, we organize our review of prior studies within the context of the DDC framework. Following this framework, Table 1 summarizes current evidence on the development of cognitive control from a time-frequency perspective. A more in-depth discussion and relevant citations can be found in the text.

**Table 1:** Summary of the development of cognitive control from a time-frequency perspective.

Note: Following the DDC framework as a guide, and focusing on time-frequency analyses of EEG, a summary of evidence for each component of cognitive control is provided (detection processes highlighted in orange, control instantiation in blue). For each, we summarize: 1) the earliest age (to date) at which relevant effects have been reported, 2) observed developmental changes, and 3) instances in which developmental phenomena have been reported exclusively for time-frequency vs. ERP approaches. A more in-depth discussion and relevant citations can be found in the text. ^1^Note that although most evidence finds increases, some studies do not find condition-specific age-related changes (Bowers et al., 2018) or report non-linear effects, especially regarding social rejection (Tang et al., 2019).

## 5 Detection processes

### 5.1 Detection processes: Novelty detection

A challenge to studying the development of cognitive control is that infants, toddlers, and young children are often unable to meet the cognitive and motor demands of experimental paradigms typically employed to study cognitive control in adults. However, one approach is to use passive listening tasks that can nonetheless assess novelty detection, a process that falls within the detection component of cognitive control (see Fig. 3). For example, a 3-stimulus oddball paradigm involves presenting a standard sound (simple tone) on most trials; a rarely presented deviant sound (simple tone of a different frequency) on some trials; and a novel sound (unique complex environmental noises) on some trials. When examining ERP responses to such paradigms, it is common to observe the mismatch negativity (MMN), which is a more negative response to the rarer deviant sound relative to the repeated standard sound (Näätänen et al., 2007). The MMN occurs around 100–250 ms after the stimulus onset over frontocentral sites and is thought to reflect automatic novelty/salience detection of the change in sound between frequent (standard) and infrequent (deviant) sounds (Näätänen et al., 2007). Additionally, the P3a is also observed when measuring ERPs in response to complex novel stimuli. The P3a is a positive deflection with midfrontal distribution that peaks around 200–400 ms post-stimulus onset (Escera et al., 1998, Marshall et al., 2009), and it is thought to reflect the detection and/or attentional orienting response towards novelty (Escera et al., 1998, Polich, 2007). In time-frequency analyses with adults, both the MMN and P3a, but especially the P3a, are associated with increased signal strength and consistency in midfrontal theta (Demiralp et al., 2001, Javitt et al., 2018, Solís-Vivanco et al., 2021). However, the development of these neural responses and their functional significance have not been well established.

We recently examined how ERP and time-frequency responses elicited by a 3-stimulus oddball paradigm change across age in a large sample of children (Morales et al., 2023). As expected, we observed an MMN in response to deviant tones and a P3a in response to novel tones, both compared to standard tones. When we examined age-related changes from 4 to 11 years, we found no changes in the MMN or P3a, in line with existing literature (Kraus et al., 1999, Riggins and Scott, 2020, Shafer et al., 2000). When conducting time-frequency analyses, we found clear frontocentral responses in theta and delta bands in response to novel tones, relative to the other conditions. Crucially, we also observed age-related increases in response to novel sounds in theta and delta power, compared to responses to other sounds (Morales et al., 2023). We also found that theta and delta consistency increased with age in response to novel sounds, compared to other sounds. These findings are in line with other studies with older children and adolescents (Bishop et al., 2011, Bishop et al., 2011). Together, these data highlight the involvement of theta (and delta) in novelty detection and that both theta signal strength and consistency in response to novelty increase with age—despite age-related effects being absent in the ERP data (Morales et al., 2023).

The minimal cognitive and motor demands of passive listening tasks (e.g., the 3-stimulus oddball) are particularly useful for studying detection processes in infants or toddlers. For instance, in a study with sleeping newborns, Isler et al. (2012) observed increased frontal theta and delta power in response to infrequent tones, compared to frequent tones. Importantly, these increases in power were evident even in the absence of an observable ERP effect (i.e., MMN). Finally, in an ongoing study, we have collected a 3-stimulus auditory oddball task in a sample of young infants between 3 and 5 months. What we observe in these data is a clear midfrontal theta response to novel sounds. We also see increases in theta consistency in the novel condition, although the topography of the consistency effect is less clear (W. Xu et al., 2023). In ongoing work, we are examining how these neural responses relate to infants’ fMRI resting state networks (Kanel et al., 2024).

The strength of passive listening tasks (low cognitive and motor demands) also becomes a limitation in terms of the ability to draw inferences as to whether observed midfrontal theta responses reflect cognitive control per se. However, this limitation can be overcome by assessing whether neural responses in passive listening tasks relate to behavioral measures of cognitive control assessed via other means. For example, in our study investigating novelty-related changes in midfrontal theta among 4- to 11-year-old children, we found that theta consistency was predictive of inhibitory control behavior in a separate task (Morales et al., 2023). This finding is in line with previous studies suggesting that theta consistency is implicated in the development of neurocognitive processes associated with inhibitory control (DuPuis et al., 2015, Gavin et al., 2019). Moreover, these data support the utility of leveraging a passive 3-stimulus oddball task to elicit neural measures associated with cognitive control, which can then be validated by assessing relations with behavior in an orthogonal task.

#### 5.1.1 Summary of novelty detection

In sum, midfrontal theta (and to some extent delta) is involved in novelty detection from infancy to adulthood. Several studies have documented developmental changes in theta signal strength and consistency. Moreover, some of these theta measures have been related to behavioral measures of cognitive control. Notably, several of these developmental changes, as well as relations with control behavior, are not observed with ERPs. These data also illustrate the potential of passive listening paradigms to examine some stimulus-driven aspects of cognitive control.

### 5.2 Detection processes: Conflict detection

Although passive listening paradigms offer a promising approach to studying stimulus-driven forms of detection involved in cognitive control development, it is not possible to examine relations with goal-directed behavior within the same task. However, utilizing a child-friendly “Go/No-Go” task, we have examined the role of theta in conflict detection and inhibitory control (X. Xu et al., 2024). In this task, children are required to respond to frequently presented stimuli and inhibit responses to infrequently presented stimuli. Accordingly, they must detect the need for control in response to infrequent stimuli and then inhibit their responses to complete this task correctly. These processes have been most studied using ERPs, including the N2 and P3. The N2 component is the second negative deflection in the waveform and occurs in children approximately 200–450 ms post-stimulus over frontal areas (Bruin and Wijers, 2002). This component has been associated with conflict detection and/or response inhibition using other Go/No-Go or similar tasks (Conejero et al., 2023, Hoyniak, 2017). However, despite several studies reporting a larger N2 for trials that require conflict detection (e.g., No-Go), recent studies with children have not found a significant change in N2 amplitudes in response to stimuli that should trigger conflict detection (Algarín et al., 2013, Andreu et al., 2024, Hosch et al., 2024, Lamm et al., 2012, Sullivan et al., 2022). This suggests important inconsistencies in the presence of the conflict-evoked N2 amplitudes in early development (Hoyniak, 2017). Of note, the N2 is typically followed by the P3 component, a positive-going wave peaking between 300 and 700 ms that is maximal over central-parietal areas (Riggins and Scott, 2020). Similar to the N2 component, the P3 is elicited by response inhibition tasks like the Go/No-go task (Huster et al., 2013). However, compared to the N2, the P3 is thought to be more closely related to control instantiation, rather than conflict detection (Polich, 2007). The P3 amplitude tends to be larger in magnitude for trials that require inhibition (No-Go) compared to stimuli that require a prepotent response (Go), starting in childhood (Conejero et al., 2023, Eimer, 1993, St. John et al., 2019). Moreover, the presence of the P3 in childhood has been more consistent than the N2 (Hoyniak, 2017, Riggins and Scott, 2020; X. Xu et al., 2024).

It is possible that mixed findings for the N2 as an index of conflict detection across development arise from confounding changes in the consistency and/or overlap of the N2 and P3 across age. Moreover, such factors might be exacerbated using child-friendly designs (e.g., use of complex animal images). Importantly, time-frequency analyses may be more robust to such confounds. Toward these ends, we recently assessed both ERP (N2/P3) and time-frequency (theta/delta) responses when children ages 4–11 years performed the child-friendly Go/No-Go task (X. Xu et al., 2024). We observed larger P3 amplitudes for No-Go (vs. Go) trials but the opposite for the N2, namely, a larger N2 (more negative magnitude) for Go (vs. NoGo) trials (X. Xu et al., 2024). However, when utilizing time-frequency analyses, we observed clear midfrontal theta and delta increases in power and consistency for trials requiring detection (No-Go trials) compared to those that did not (Go trials). This is in line with literature in adults, which not only suggests that the N2 and midfrontal theta are implicated in detection, but that the N2 and P3 reflect a combination of distinct but overlapping processes that can be better indexed with time-frequency analyses (Harper et al., 2014, Harper et al., 2016). Moreover, when looking at age-related changes between 4 and 11 years, we found that theta and delta power during inhibition trials increased and had a shorter latency to peak with age, compared to non-inhibition trials (X. Xu et al., 2024), consistent with other work in children and adolescents finding similar power and latency changes with age (Adam et al., 2020, Liu et al., 2014).

There is evidence for the role of theta in conflict detection in other contexts, including in younger children. For example, toddlers and infants display increases in midfrontal theta to conflicting information or expectancy violations in the environment, like puzzles or arithmetic problems being solved incorrectly (Berger et al., 2019, Berger and Posner, 2023, Conejero et al., 2018). Of note, we broadly refer to such studies as reflecting conflict detection as they can be described as involving situations in which conceptual representations or expectancies conflict with one another. Moreover, the experimental paradigms employed are such that the results cannot easily be explained by simple differences in stimulus characteristics or probability. That is, incorrect solutions occurred at an equal or greater rate than correct solutions, stimuli were often repeated, and stimuli with clear perceptual differences were excluded in some studies. These factors support the interpretation that infants were responding to conceptual violations of expectation as opposed to novelty.

In a study by Conejero and colleagues (2018), toddlers observed puzzles being completed either correctly or incorrectly. Although the ERP responses differed considerably in timing and morphology between infants and adults, both groups displayed a similar midfrontal theta response—albeit the adult response to incorrect, relative to correct solutions, was stronger (Conejero et al., 2018). Similarly, Berger et al. (2019) reported increased midfrontal theta power in 6- to 8-month-old infants to prediction errors in the context of arithmetic problems using a variation of the classic violation of expectation paradigm by Wynn (1992). In a similar manner to adults, increases in midfrontal theta have also been reported to action sequences completed in unexpected ways, starting at 9 months (Reid et al., 2009). Moreover, Köster and colleagues (2019, 2021) found that 9-month-old infants displayed increased frontal theta to several unexpected events (e.g., actions, numbers, solidity), implying that frontal theta serves as a domain-general mechanism to index prediction error from the first year of life. Furthermore, by using visual entrainment, they showed that these responses to prediction error/conflict were specific to the theta band (Köster et al., 2019). Finally, Köster et al. (2021) also showed that these theta responses were relatively independent of ERP responses, highlighting the complementary value of time-frequency measures and time-domain (ERP) approaches.

#### 5.2.1 Summary of conflict detection

In summary, prior studies employing behavioral paradigms in children show that, similar to adults, children as young as four display midfrontal theta and delta increases in power and consistency (ITPS) in response to conflicting stimuli that require inhibition. Moreover, these midfrontal theta/delta power responses increase in magnitude and decrease in latency across childhood and adolescence (Adam et al., 2020, Liu et al., 2014; X. Xu et al., 2024). Other studies find that when infants or young children are presented with situations in which conceptual representations or expectancies conflict with one another, increased midfrontal theta is similarly observed (Berger and Posner, 2023). These latter studies find that midfrontal theta may be implicated in detecting conflict as early as the second half of the first year of life (Berger et al., 2019, Köster et al., 2019, Köster et al., 2021). In subsequent sections, we turn to studies examining children’s responses to receiving feedback and detecting their own errors as additional examples of the detection component of cognitive control.

### 5.3 Detection processes: Feedback detection

When engaged in goal-driven behavior, feedback (positive or negative) can provide crucial information as to whether current actions/outcomes are consistent with achieving one’s goals. Accordingly, negative feedback is assumed to serve as a signal that control is needed to change behavior and/or update one’s model of the task/world (Walsh and Anderson, 2012). The most studied feedback-related ERP is a difference wave composed of the difference between the Feedback-Related Negativity (FRN) and Reward Positivity (RewP), which are maximal over frontocentral scalp regions approximately 250–350 ms after the presentation of negative (loss/punishment) and unexpected positive (win/reward) feedback, respectively (Holroyd et al., 2008, Miltner et al., 1997). Most studies have focused on examining this difference wave in monetary gambling or probabilistic learning tasks, but studies have also examined responses to social acceptance/rejection using both ERPs and time-frequency analyses (Crowley et al., 2009, Kujawa et al., 2014, Morales et al., 2019, Tang et al., 2019). The studies examining time-frequency analyses of feedback processing find midfrontal theta and delta responses (Van der Molen et al., 2017), starting in early childhood (Crowley et al., 2014, Morales et al., 2019, Tang et al., 2019, van Noordt et al., 2015).

Although the FRN/RewP has been measured in young children, adolescents, and adults, its development remains unclear, with some studies finding increases (Hämmerer et al., 2011), decreases (Arbel et al., 2018), or no age-related changes (Kujawa et al., 2018, Lukie et al., 2014). Similarly, in a recent study of adolescent girls, we observed typical win vs. loss differences but no age-related changes in feedback-related ERPs (Bowers et al., 2018). However, when examining the same data using time-frequency analyses, we observed greater midfrontal theta responses in terms of both signal strength (power) and consistency (ITPS) for losses, relative to wins. Importantly, we also elucidated significant age-related changes in midfrontal theta and delta for both signal strength (power) and consistency (ITPS; Bowers et al., 2018). Other groups have also described greater midfrontal theta and delta responses for negative feedback, compared to positive feedback, in children and adolescents (Arbel et al., 2018, Gul et al., 2023, Gul et al., 2024, Tang et al., 2019, van Noordt et al., 2015), as well as age-related differences (Crowley et al., 2014, Tang et al., 2019). For example, Crowley et al. (2014) also identified feedback-related increases in midfrontal theta power and consistency from childhood to adolescence. Interestingly, this latter study found that the difference in midfrontal theta consistency for reward vs. non-reward feedback emerged later in adolescence and was not present in children 10–12 years old. This result is consistent with findings from other tasks that also suggest the emergence of condition differences in theta consistency are not evident earlier in childhood (Morales et al., 2022, Morales et al., 2023; X. Xu et al., 2024). Although, to our knowledge, there are no developmental studies in early childhood examining feedback detection, studies have reported midfrontal theta power responses to negative feedback (social rejection) in children as young as age 4 (Morales et al., 2019). Specifically, Morales et al. (2019) found that within a sample of 4- to 8-year-olds, midfrontal theta power was increased in response to feedback indicating rejection vs. acceptance by a hypothetical peer—an effect that did not change with age within the sample studied. Crucially, future studies should examine the role of midfrontal theta in feedback detection in younger children, as it currently remains unknown whether similar phenomena would be observable in toddlerhood or even infancy.

#### 5.3.1 Summary of feedback detection

Collectively, current evidence supports the involvement of midfrontal theta in feedback detection starting in early childhood. Moreover, these theta responses to feedback seem to increase with age during childhood and adolescence, and several of these differences do not seem evident with ERP approaches. Finally, midfrontal theta power seems to play a role in feedback detection from early childhood, but future investigations are needed to establish its role in infancy and toddlerhood.

### 5.4 Detection processes: error detection

Whereas feedback detection relies on external information to indicate whether actions/outcomes are consistent with achieving task goals, error detection (error monitoring) refers to the self-detection of actions that deviate from goal-directed behavior. Accordingly, detecting that one has committed an error can serve as a signal that control is needed to reduce the likelihood of committing future errors (Rabbitt, 1966). Most studies investigating error detection/monitoring focus on the Error-Related Negativity (ERN) and the Error Positivity (Pe). The ERN is a negative midfrontal deflection maximal immediately following an error response (0–100 ms), which is thought to reflect the rapid, automatic, and likely unconscious detection of errors (Falkenstein et al., 1991, Gehring et al., 1993). The Pe is a midparietal positive deflection maximal 200–500 ms following an error response (Falkenstein et al., 2000), thought to reflect a slower, more deliberative process that involves the conscious awareness of having made an error (Boldt and Yeung, 2015, Nieuwenhuis et al., 2001, Overbeek et al., 2005, Steinhauser and Yeung, 2010).

Studies examining age-related changes in error-related ERPs from late childhood through adolescence generally find that the ERN and Pe increase in magnitude with age during this developmental window (Buzzell et al., 2017, Davies et al., 2004, Gavin et al., 2019, Ladouceur et al., 2007, Tamnes et al., 2013). Studies of error-related ERPs in younger children suggest a similar trend (DuPuis et al., 2015, Torpey et al., 2012), although the evidence is more equivocal (Grammer et al., 2014, Lo et al., 2015). In line with these latter findings, in a large sample of 4- to 9-year-old children, we recently observed both an ERN and Pe in response to errors, but a lack of age-related changes across the sample (Morales et al., 2022). When examining time-frequency results for the same data, we observed clear midfrontal theta and delta increases in signal strength (power) and consistency (ITPS) in response to errors (vs. correct) trials. Critically, we found that error-related theta and delta power increased with age from 4 to 9 years. In terms of age-related changes in signal consistency, we observed increases in midfrontal delta, but not theta. It is worth noting that two other studies have also found evidence for age-related increases in error-related signal consistency (DuPuis et al., 2015, Gavin et al., 2019), albeit in the theta band as opposed to the delta band. Future work is needed to determine if differences across studies arise from the specific task paradigm employed, sample characteristics, or differences in cross-sectional vs. longitudinal measurement. Nonetheless, the results of these studies emphasize the utility of employing time-frequency analyses of EEG, and the results are broadly consistent in terms of observing age-related increases in the consistency of slower (<8 Hz) error-related oscillations recorded over midfrontal cortex.

#### 5.4.1 Summary of error detection

Collectively, these findings highlight three important implications. First, the results on error monitoring reflect another example of time-frequency analyses elucidating age-related differences in cognitive control phenomena not captured with ERP analyses. Second, the studies reviewed generally suggest the presence of important changes in error-related theta power (strength) and especially theta/delta ITPS (consistency) from early childhood through adolescence (DuPuis et al., 2015, Morales et al., 2022) and into adulthood (Gavin et al., 2019). Finally, in line with the more extensive adult and adolescent work, the results of Morales et al. (2022) suggest the involvement of midfrontal theta (and delta) power and consistency when young children make an error, as early as four years, regardless of age-related changes. Although the ERN has been reported with children as young as three, to our knowledge, four years is the youngest age that error-related increases in midfrontal theta have been documented to date. It seems plausible that midfrontal theta serves as a mechanism for the self-detection of errors in children younger than four, perhaps even as early as the first year of life. As discussed above (Section 4.2 on conflict detection), there is evidence that infants as young as six months can detect incorrect solutions to arithmetic problems or puzzles, with commensurate increases in event-related midfrontal theta (Berger et al., 2019, Berger and Posner, 2023). However, although such paradigms examine midfrontal theta elicited by prediction errors arising from sensory information in the environment, this is distinct from the self-detection of errors arising from one’s own actions. Thus, future research using novel paradigms is needed to examine whether it is possible to observe event-related increases in midfrontal theta based on the self-detection of one’s own errors (error monitoring).

## 6 Control instantiation

As previously discussed, cognitive control can be conceptualized as consisting of at least two components, the first being detection that control is needed, and the second being control instantiation. To provide evidence that a given neural process is associated with detection, it is minimally sufficient to contrast neural activity arising in response to events signaling the need for control (e.g., deviant stimuli, error responses) with events that do not (e.g., standard stimuli, correct responses). However, to provide evidence that a given neural process is associated with control instantiation requires either demonstrating engagement of neural regions previously linked to control instantiation or demonstrating a positive association with controlled behavior (e.g., behavior indicative of the inhibition of responses, increased attention, updating/manipulating working memory). Both approaches have limitations. Drawing inferences based solely on the engagement of neural regions previously linked to control instantiation is subject to the reverse-inference problem (Poldrack, 2006). Similarly, relying only on the observation that a neural process is positively associated with controlled behavior is limited by the fact that detection is also often predictive of control, given that detection processes typically precede control instantiation. Therefore, any simple association between a given neural measure and controlled behavior could be indicative of either control instantiation, or an upstream detection process (that then leads to control instantiation). At a measurement level, the only way to fully address this latter issue is to employ statistical analyses to demonstrate whether a given measure uniquely predicts control behavior above and beyond other neural processes. However, there are only limited examples of such approaches being applied in the literature, especially for pediatric studies of cognitive control. Instead, most evidence for control instantiation relies on either drawing inferences based on the engagement of neural regions previously linked to control, simple pairwise associations with control behavior, or some combination of these approaches. We view such approaches as providing at least initial evidence consistent with the possibility that a given neural process reflects control instantiation. Nonetheless, it is important to consider these methodological limitations when reviewing the literature.

Prior work in adults suggests the medial-frontal cortex (MFC) is primarily engaged in the detection that control is needed, whereas the instantiation of control requires recruitment of control regions (e.g., lateral-frontal cortex) to direct the instantiation of control in a task-relevant manner^5^ (Gratton et al., 2018, Kerns et al., 2004, Ridderinkhof et al., 2004). Following the detection that control is needed via the MFC, communication with lateral-frontal (or other) control regions to instantiate control is thought to rely, at least in part, on the alignment/synchronization of theta oscillations between brain areas (Cavanagh and Frank, 2014). In terms of time-frequency measures, this can be indexed by computing the alignment/synchronization of theta oscillations for electrodes located over distinct cortical regions, a measure referred to as inter-channel phase synchrony (ICPS) that is sometimes interpreted heuristically as a measure of “connectivity” (i.e., reflecting neural communication) between two brain regions (Morales and Bowers, 2022). The rationale behind this measure is that if two neural regions rely on a given oscillation to communicate (e.g., aligning states of excitation/inhibition of local neural ensembles), then the oscillations recorded at nearby electrodes should be synchronized, indexing that they are functionally connected (Cavanagh and Frank, 2014, Fries, 2005, Uhlhaas and Singer, 2010). Consistent with this view and the proposed role of event-related theta oscillations in cognitive control, prior work in adults demonstrates that the alignment/synchronization of theta oscillations between electrodes located over MFC and frontal-lateral control regions are predictive of behavioral adaptations indicative of control instantiation (reviewed in Cavanagh and Frank, 2014). However, only limited work has tested similar phenomena reflecting control instantiation in youth.

In one of the few pediatric investigations, we found that alignment/synchrony of theta oscillations between midfrontal and frontolateral electrodes (“connectivity”) was implicated in various forms of control instantiation within a sample of adolescents (Buzzell et al., 2019). Participants performed a flanker task, in which rapid responses must be made to stimuli that sometimes involve conflicting information (Eriksen and Eriksen, 1974). We observed that midfrontal-frontolateral theta connectivity that occurred prior to making a response, and which was triggered by the presentation of stimuli involving conflict, was associated with making a correct response on the current trial—suggesting involvement in control instantiation associated with the resolution of conflict. We also observed that error responses were immediately followed by increases in theta power and synchrony at midfrontal electrodes, as well as increases in midfrontal-frontolateral theta connectivity. Moreover, theta connectivity between midfrontal electrodes and more rostral/caudal frontolateral locations predicted distinct forms of behavioral control in post-error trials. Crucially, we found that midfrontal-frontolateral theta connectivity fully mediated the degree to which increased theta signal strength (power) and consistency (ITPS) predicted post-error behavioral adaptations (Buzzell et al., 2019). Similar findings recently reported increased theta connectivity between midfrontal and frontolateral locations when utilizing feedback effectively in children and adolescents (Gul et al., 2024). In sum, these data provide strong evidence consistent with the notion that, at least among adolescents, MFC theta power and synchrony are more closely associated with the detection that control is needed, whereas theta connectivity between MFC and distinct control regions are more closely associated with distinct forms of control instantiation.

In the only study with young children, to our knowledge, we examined midfrontal-frontolateral connectivity in a large sample of children who completed a child-friendly Go/No-Go task (Morales et al., 2022). First, we found increased connectivity after errors between midfrontal and frontolateral electrodes in the theta and delta bands across all children. Moreover, when examining age-related changes, we found increases in connectivity from 4 to 9 years in the delta band—the theta band showed a similar increasing pattern, but it was not significant. This suggests that the connectivity between midfrontal and frontolateral regions develops across early childhood. Although we did not also assess relations with controlled behavior in this study, these data are generally consistent with the notion that, starting in early childhood, theta and delta oscillations may serve as a neural mechanism by which the error-monitoring system signals the need for increased control and allows for communication between brain regions after errors. Moreover, time-frequency measures of connectivity in this error monitoring system can be used to index development across childhood (Morales et al., 2022). However, additional work is needed to assess whether/how these measures directly relate to controlled behavior in children. Moreover, future studies are needed to examine if similar connectivity patterns are present in infants and toddlers.

Distinguishing between detection and control instantiation in younger children and infants is more challenging and, to our knowledge, has not been done in part due to the motor and cognitive demands of most cognitive control paradigms. Nonetheless, findings consistent with theta supporting control instantiation processes have been observed as early as infancy. First, a set of studies have shown that infant sustained attention, as defined by heart rate deceleration during the presentation of engaging videos, was associated with increased frontal theta power (Brandes-Aitken et al., 2023, Xie et al., 2018). Moreover, this effect increased across infancy from 6 to 12 months, suggesting the involvement of theta power in sustained attention emerges around 10–12 months (Xie et al., 2018). However, recent studies have reported increased frontal theta power during sustained attention in infants as young as three months (Brandes-Aitken et al., 2023). Notably, Xie et al. (2018) distinguished between periods of attention orienting, sustained attention, and attention termination, finding that increases in theta were especially prominent during sustained attention and that these effects were source localized to frontal and ventral regions (e.g., orbitofrontal cortex). This implies that frontal theta can be involved in control processes (i.e., sustained attention), rather than just detection processes (i.e., orienting) in infancy.

Evidence for the involvement of theta and control instantiation in infants also comes from live interaction studies. For example, some of the first studies on infant theta found increases in frontal theta power for anticipatory attention during a live game of peekaboo during the second half of the first year (Orekhova et al., 1999, Stroganova et al., 1998). Moreover, the authors found that the magnitude of frontal theta power was related to the extent of infants’ anticipatory attention behavior (Orekhova et al., 1999, Stroganova et al., 1998). The authors interpreted this as frontal theta reflecting endogenously controlled attention, as frontal theta was not increased during exogenously controlled attention (i.e., when a person distracted the infant with bubbles). Thus, we would similarly interpret these findings as an early form of control instantiation, and specifically as a form of planful control engaged in a proactive manner in expectation of a future event. Similarly, in a more recent study, Wass et al. (2018) found that infant theta power while playing with toys predicted subsequent increased attention (i.e., longer looks) to the toys. Importantly, the authors found that this was particularly true when infants engaged in solo play, rather than when interacting with adults, suggesting that theta is implicated in endogenous attention rather than other exogenous forms of attention (e.g., parent-directed play; Wass et al., 2018). These findings highlight the role of frontal theta in goal-driven, planful forms of control instantiation starting as early as infancy.

Additional, indirect evidence for the role of theta in control instantiation comes from studies of frontal theta power during moments of heightened attention that support active learning. For example, during social interactions, ostensive cues, or infant-directed speech (Michel et al., 2024; Orekhova et al., 2006; Zhang et al., 2011; see Begus and Bonawitz, 2020 for a review). For instance, increased midfrontal theta power during mother-infant interactions has been recently shown to predict better infant learning (Michel et al., 2024). Finally, although not linked to cognitive control in the moment as we have been discussing for most studies in this review, emerging evidence has linked frontal theta power in infants while viewing complex videos with future non-verbal intelligence and attention control months to years later (Braithwaite et al., 2020, Hendry et al., 2023, Jones et al., 2020). Although not tied to specific cognitive control processes, this evidence highlights the role of measures of frontal theta in helping understand the mechanisms of infant attention from the first years of life, as well as serving as an early indicator of later cognitive ability.

### 6.1 Summary of control instantiation

In summary, multiple sources of evidence support the involvement of midfrontal theta in control instantiation across development. There is strong evidence that theta connectivity between midfrontal and frontolateral electrodes is associated with control instantiation in adolescents. Moreover, data in children is also generally consistent with such midfrontal-frontolateral theta connectivity being associated with control instantiation. However, to our knowledge, studies in infants and young children have not tested for similar associations between control instantiation and midfrontal-frontolateral theta connectivity. Nonetheless, several studies have shown that midfrontal theta power is associated with sustained attention and endogenously controlled attention as early as infancy. Similarly, studies find that frontal theta is involved in heightened attention and learning. Future studies are needed to better understand the connections between these different sources of evidence and examine developmental changes in control instantiation.

## 7 Associations between midfrontal theta and individual differences in behavior, early risk factors, and psychopathology

Although the focus of this review is on normative development, in the final section of the review, we highlight how midfrontal theta relates to individual differences in behavior, as well as emerging studies investigating associations with early life experiences and psychopathology. First, midfrontal theta, as a mechanism of cognitive control, not only has been implicated in condition differences (e.g., congruent vs. incongruent or error vs. correct) at the group level (e.g., children vs. adults), but has also been linked with individual differences in behavioral measures of cognitive control. For example, midfrontal theta consistency in response to auditory novelty was related to accuracy in an inhibitory control task (Morales et al., 2023). Similarly, conflict-related theta power and latency have been related to faster reaction times (Adam et al., 2020; X. Xu et al., 2024) and cognitive control behavior (Liu et al., 2014; X. Xu et al., 2024). Feedback-related delta signal strength, but not ERPs, have been associated with task accuracy and learning outcomes (Gul et al., 2023). Error-related theta power and consistency have been related to behavioral measures of cognitive control, including shorter RT, decreased RT variability, increased post-error slowing, and increased task accuracy (Buzzell et al., 2019, DuPuis et al., 2015, Pietto et al., 2023).

Moving beyond proximal associations between midfrontal theta and individual differences in task behavior, cognitive control is considered a fundamental component of emotion regulation and self-regulation more broadly (Nigg, 2017, Ochsner et al., 2012, Ochsner and Gross, 2005). This has led to research investigating empirical relations with several forms of psychopathology, supporting claims that cognitive control is one of the strongest transdiagnostic risk factors (Cavanagh and Shackman, 2015, McLoughlin et al., 2022). Importantly, emerging studies suggest that the association between midfrontal theta and broad dimensions of psychopathology is also present in childhood and adolescence (Buzzell et al., 2020, Dell’Acqua et al., 2023a, Dell’Acqua et al., 2023b, Michelini et al., 2022; X. Xu et al., 2024). Given that cognitive control ability is one of the most notable transdiagnostic risk factors (Martel et al., 2017, McTeague et al., 2017, Robson et al., 2020, Snyder et al., 2017), specific links between midfrontal theta and transdiagnostic risk are also generally consistent with the notion that midfrontal theta is a putative mechanism of cognitive control.

Given the relevance of frontal theta to cognitive control and risk for psychopathology, several studies have turned to understanding the early contextual predictors of frontal theta. This includes relations with family socioeconomic status (Conejero et al., 2018; X. Xu et al., 2024) and prenatal risk factors such as prenatal alcohol exposure (Berger et al., 2019). For example, one of the first studies examining these relations found that conflict-related theta in toddlerhood was predicted by parental SES, such that increased SES predicted increased theta power in response to events involving increased conflict (i.e., watching a puzzle being solved incorrectly) (Conejero et al., 2018). Similarly, we aimed to replicate and extend these relations by examining the role of prenatal SES, theta power elicited by conflict, and relations to child concurrent psychopathology (X. Xu et al., 2024). We found that children of mothers with higher SES during pregnancy displayed increases in midfrontal theta power. Moreover, we found that conflict-related theta power, in turn, was associated with fewer externalizing problems. Finally, we found support for mediation, implying that midfrontal theta power may serve as a developmental pathway linking early risk factors to later behavioral and emotional problems in children (X. Xu et al., 2024).

The findings reviewed above are closely in line with another study in which we investigated more extreme forms of early deprivation by utilizing data from the Bucharest Early Intervention Project (BEIP)—a longitudinal, randomized-control trial examining the effects of early institutionalization and a high-quality foster care intervention among children in Romania (Nelson et al., 2014). In line with animal models of neglect (Courtiol et al., 2018, Sarro et al., 2014), children who experienced early neglect due to institutional rearing exhibited lower event-related midfrontal theta in adolescence (Buzzell et al., 2020). Moreover, children who were placed into the foster care intervention at a younger age showed greater midfrontal theta power. Finally, early foster care placement predicted a developmental trajectory of midfrontal theta across the adolescent period (ages 12–16), which mediated decreases in general psychopathology across the same period (Buzzell et al., 2020). To our knowledge, this study provides the only experimental evidence that midfrontal theta is impacted by early neglect in humans, replicating similar findings from animal models of early adversity. In this way, midfrontal theta can help us understand how early experiences are associated with later cognitive and socioemotional development. Collectively, these findings reinforce the importance of applying time-frequency analyses to EEG data to study the role of oscillations in the development of cognitive control, as well as to understand factors influencing these phenomena.

## 8 Recommendations for future research

As discussed in this article, there is emerging evidence suggesting that midfrontal theta is a mechanism of cognitive control from early development. Yet, there are important limitations to our current knowledge that could be addressed by future research. Most of the studies reviewed, and all studies in infancy and early childhood, were cross-sectional. There is a need for more longitudinal studies to examine developmental trends that may be obscured by cross-sectional data and to understand the stability of rank-order associations in infancy and early childhood. Additionally, there is a need to create new paradigms that allow examining the role of midfrontal theta in feedback detection and error detection, as well as control instantiation, in infancy and toddlerhood. Currently, we rely on computerized tasks that are not amenable for young children. We will likely need more live, interactive paradigms, which would also increase ecological validity.

Of the studies reviewed, it is worth emphasizing that similar developmental patterns seem to be present for oscillations in the theta and delta bands, although associations with behavior and psychopathology seem more consistent (and almost exclusively found) with the theta band. Several studies examine midfrontal theta a priori without considering other frequency bands and topography. It is important for future research to examine the specificity of midfrontal theta by examining effects at other frequency bands and locations. Indeed, although the current review has focused on the role of theta in cognitive control, evidence in adults also links aspects of cognitive control with other frequency bands as well (Helfrich and Knight, 2016). One area of particular interest is findings from adult studies demonstrating that power/phase relations across distinct frequency bands (cross-frequency coupling), typically involving a relatively slower band (theta/delta) and a relatively faster band (gamma), are central to cognitive control (Helfrich and Knight, 2016). A second area of interest is emerging evidence from adult studies that point to a putative role of beta bursts in cognitive control (Lundqvist et al., 2024). However, these phenomena remain largely unexplored in pediatric samples or across development.

In addition to prior developmental studies focusing on relatively “narrow band” analyses of theta in cognitive control, these studies have not considered the possibility of broader shifts in the event-related power spectrum (i.e., aperiodic slope, offset: Donoghue et al., 2020). Thus, it remains entirely unknown whether developmental associations between event-related theta and cognitive control are indicative of broader shifts in the power spectrum relating to cognitive control. For example, it is possible that reports of age-related increases in event-related theta, and commensurate associations with cognitive control, could at least partially be explained by event-related increases in broadband power (increases in aperiodic offset) or indicate a broader event-related pattern of increased lower-frequency and decreased higher-frequency power (i.e., increased aperiodic slope). However, these possibilities remain untested, given that studies investigating age-related changes in aperiodic slope/offset in youth have focused on resting state data (for a review, see Stanyard et al., 2024) as opposed to within cognitive control tasks (for a review of how resting resting-state theta relates to cognitive function across development, see Tan et al., 2024). Emerging work in adults has begun to examine potential event-related changes in aperiodic slope/offset within cognitive control tasks (e.g., Jia et al., 2024; Lu et al., 2024; C. Zhang et al., 2023)—future work should conduct similar investigations in youth. Importantly, if future work finds that age-related increases in event-related theta can at least partially be explained by broader (aperiodic) changes in the power spectrum, this would not necessarily supplant the conclusions drawn in this review. However, such findings would provide a broader empirical and theoretical context (Donoghue et al., 2020) within which to interpret associations between age-related increases in event-related theta and cognitive control, providing a bridge to understanding how the balance of excitatory/inhibitory neural activity relates to cognitive control.

Despite the advantages of time-frequency approaches outlined in this review, there are also limitations to consider, consistent with the notion that time-frequency approaches should ultimately be viewed as complementary to traditional approaches like ERPs. Although a strength of time-frequency analyses is their sensitivity to non-time-locked effects, this can also lead to a limitation insofar as time-frequency methods may be more susceptible to artifacts relative to time-locked methods (e.g., ERPs). Given that artifacts may potentially be more prevalent in pediatric EEG data, it is crucial that researchers interpret results cautiously. The timing, topography, and spectral characteristics of many common artifacts have been well-characterized (Keren et al., 2010, Leach et al., 2020, Mognon et al., 2011, Muthukumaraswamy, 2013, Pion-Tonachini et al., 2019, Plöchl et al., 2012), which can facilitate researchers carefully considering whether observed time-frequency results are confounded by artifacts. The current review focuses on effects within the theta and delta range with a midfrontal topography; to our knowledge, these effects are less likely to be impacted by known artifacts. Moreover, most of the findings described in the current review are baseline corrected and in comparison to another condition (e.g., Go vs. No-Go, Error vs. Correct, etc.). Focusing on condition-specific effects can also serve to guard against potential confounds arising from non-specific artifacts—although one must still consider the possibility that certain artifacts are correlated with conditions of interest. Finally, researchers should look for convergent evidence across different methods and disciplines, especially those that may be less affected by artifacts (e.g., as discussed above, midfrontal theta has been extensively studied in adults, animal models, and invasive recordings). Given the relative novelty of applying time-frequency EEG methods to study early development, future research utilizing these approaches should take care not to overinterpret potential artifacts as neural effects.

Emerging evidence highlights the influence of context across levels of analysis on midfrontal theta. For example, emerging work demonstrates that midfrontal theta dynamics are impacted by the local social context (e.g., presence of a peer, caregiver, or parent-child interactions; Buzzell et al., 2019), as well as the broader socioemotional developmental context (e.g., caregiving environment, adversity, and culture; Buzzell et al., 2020; Conejero et al., 2018; Xu et al., 2024). However, we are still only beginning to understand the role of midfrontal theta in social information processing, social learning, and the impact of early life experiences. Future studies are needed to understand how midfrontal theta interacts with, and is shaped by context, and its potential role(s) in developmental pathways linking early experiences to future outcomes (e.g., psychopathology).

Finally, it is important to note that the DDC framework (Fox et al., 2021, Fox et al., 2022) was employed in the current review as a heuristic to facilitate organizing a description of cognitive control phenomena. As such, this framework was not meant to serve as a model of the development of cognitive control. As data continues to accumulate on the role of oscillations in the development of cognitive control, theoretical work is critically needed to develop a comprehensive model that can not only accurately describe the development of cognitive control, but also serve to make novel, testable predictions. Similarly, our review emphasizes the putative role of developmental changes in midfrontal theta in the emergence of cognitive control. Consistent with the views advanced by others, we suggest that theta oscillations support cognitive control by providing the temporal structure necessary for the integration/segregation of information within/between distributed brain networks involving the MFC (Cavanagh and Frank, 2014, Cohen, 2014a, Duprez et al., 2020, Helfrich and Knight, 2016). This view is supported by biophysical principles describing how oscillations in the local field potential impact the neuronal firing within and across cortical regions (Buzsaki, 2004, Buzsáki et al., 2012, Fries, 2005). Nonetheless, there is a critical need for these ideas to be formalized as computational models that predict the emergence of cognitive control from developmental changes in midfrontal theta oscillations. The studies reviewed can provide the foundation to inform such models.

## 9 Conclusion

Midfrontal theta appears to reflect a neural mechanism supporting cognitive control across development, starting in infancy. As suggested by the studies reviewed, midfrontal theta is implicated in several cognitive control processes associated with both detecting the need for control and control instantiation. Moreover, midfrontal theta shows important developmental changes in terms of both power and phase dynamics, which closely align with the development of cognitive control abilities. For most tasks/contexts studied, midfrontal theta signal strength and consistency broadly increase across development. This developmental pattern is evident across domains and aspects of cognitive control, including the detection of novelty, conflict, feedback, and errors. Importantly, in many of the cognitive control studies reviewed, we do not consistently observe corresponding developmental changes when employing ERP-based measures. Similarly, identified relations between midfrontal theta and other individual differences in early life experiences or psychopathology are not always mirrored in ERPs, again, highlighting the added utility of studying brain oscillations via time-frequency analyses.

Collectively, the reviewed data are consistent with the notion that midfrontal theta may serve as a mechanism of cognitive control. Critically, a direct read-out of this putative neural mechanism can be non-invasively measured across development via EEG and time-frequency analyses. Notwithstanding the value of ERP approaches, we emphasize the importance of performing time-frequency analyses to extract additional, valuable information from developmental EEG data. To encourage the broader adoption of time-frequency analyses within the developmental neuroscience community, we have provided accessible tutorials, which we recommend exploring for further insights into these methods (Buzzell et al., 2022, Morales and Bowers, 2022; also see Buzzell et al., 2023).

## CRediT authorship contribution statement

**Santiago Morales**: Writing – review & editing, Writing – original draft, Conceptualization. **George A. Buzzell**: Writing – review & editing, Conceptualization.

## Funding

SM was supported by grants from the 10.13039/100000002National Institutes of Health (UH3 OD023279) and the 10.13039/100000865Bill and Melinda Gates Foundation (INV-047884). GB was supported by grants from the National Institute of Mental Health of the National Institutes of Health under award numbers R01MH131637 and R21MH131928. Contents of the current report are the sole responsibility of the authors and do not necessarily represent the official views of the funders.

## Declaration of Competing Interest

The authors declare that they have no known competing financial interests or personal relationships that could have appeared to influence the work reported in this paper.

## Data Availability

No data was used for the research described in the article.

## Footnotes

2 Cognitive control is closely related to several other constructs such as attentional control, executive function(s), effortful control, and self-regulation. Note that these constructs can be at least partially overlapping, and the use terminology used in the literature is not always consistent. For the purposes of this review, we use the term “cognitive control” to refer to a set of rapidly occurring processes that can be divided into two broad components, including processes that detect the need for control and processes that implement control to achieve goal-directed behavior. For a detailed taxonomy and discussion of cognitive control and other related constructs, see Nigg (2017).

3 Although time-frequency measures are more forgiving of imprecise timing in tasks and associated cognitive processes, researchers must interpret results with caution. Namely, the sensitivity of time-frequency measures to non-time-locked activity also leads to possible susceptibility to artifacts. See “Recommendations for Future Research” for further discussion on this potential limitation.

4 It is important to distinguish between the largely non-overlapping findings associated with analyses of event-related theta (i.e., oscillations elicited in response to a task event) and resting state theta (i.e., oscillations when the brain is not engaged in an explicit task). Event-related theta and resting state theta are often associated with early environmental experiences, executive functions, and psychopathology in opposing directions (Tan et al., 2024). The current review focuses exclusively on event-related theta in relation to cognitive control.

5 Note that there are exceptions to the broad view that control instantiation requires coordination with brain regions outside the MFC (e.g., lateral-frontal cortex). For example, the Cascade of Control model (Banich, 2009) proposes the MFC is capable of not only detecting but also instantiating control. Similarly, Petersen and Poster (2012) propose a model of attention where, in addition to control instantiation largely being engaged via frontal/parietal regions, subregions of the MFC play a role in maintaining control instantiation over time. However, such frameworks/models are beyond the scope of the current review.
